# *Mobility Rehab* visual feedback system for gait rehabilitation in older adults

**DOI:** 10.1186/s12984-023-01260-2

**Published:** 2023-10-24

**Authors:** Carla Silva-Batista, Graham Harker, Rodrigo Vitorio, Mike Studer, Brady Whetten, Jodi Lapidus, Patricia Carlson-Kuhta, Sean Pearson, Jess VanDerwalker, Fay B Horak, Mahmoud El-Gohary, Martina Mancini

**Affiliations:** 1 Department of Neurology, Oregon Health & Science University, 3181 SW Sam Jackson Park Rd, OP-32, Portland, OR 97239 USA; 2 Northwest Rehabilitation Associates, Salem, OR USA; 3 Biostatistics and Design Program Core, Oregon Health & Science University, Portland, OR USA; 4 APDM Wearable Technologies – a Clario Company, Portland, OR USA

**Keywords:** Older adults, Mobility training, Sensor-based feedback, Wearable inertial sensors, Parkinson’s disease, Outpatient clinic

## Abstract

**Background:**

Gait and balance impairments are among the main causes of falls in older adults. The feasibility and effectiveness of adding sensor-based feedback to physical therapy (PT) in an outpatient PT setting is unknown. We evaluated the feasibility and effectiveness of PT intervention combined with a therapist-assisted visual feedback system, called *Mobility Rehab*, (PT + MR) in older adults.

**Methods:**

Twenty-eight older adults with and without neurological diseases were assigned either PT + MR (n = 22) or PT alone (n = 6). Both groups performed 8 sessions (individualized) of 45 min long (30 min for gait training and 15 min for endurance, strength, and balance exercises) in an outpatient clinic. *Mobility Rehab* uses unobtrusive, inertial sensors on both wrists and feet, and at the sternum level with real-time algorithms to provide real-time feedback on five gait metrics (step duration, stride length, elevation at mid-swing, arm swing range-of-motion [ROM], and trunk coronal ROM), which are displayed on a tablet. The primary outcome was the Activities-specific Balance Confidence scale (ABC). The secondary outcome was gait speed measured with wearable inertial sensors during 2 min of walking.

**Results:**

There were no between-group differences at baseline for any variable (P > 0.05). Neither PT + MR nor PT alone showed significant changes on the ABC scores. PT + MR, but not PT alone, showed significant improvements in gait speed and arm swing ROM. The system was evaluated as ‘easy to use’ by the PT.

**Conclusions:**

Our preliminary results show that PT + MR improves gait speed in older adults with and without neurological diseases in an outpatient clinic.

**Clinical Trial Registration:**

www.ClinicalTrials.gov, identifier: NCT03869879.

## Introduction

Gait and balance impairments often lead to falls in older adults and people with neurological diseases (e.g., Parkinson’s disease and stroke) [1–4]. Gait impairments often manifest as slow gait with increased double support time, reduced stride length, shuffling, and decreased arm swing and turning velocity [3, 5–9]. These deficits in upper and lower body gait metrics, that are usually multifactorial in origin [10], require a comprehensive assessment to identify the risk of falling and to target intervention for older adults and people with neurological diseases.

Although physical therapists observe patients’ walking patterns and provide verbal and/or somatosensory feedback to improve their patients’ mobility, these methods are not optimal because clinical gait observation is subjective, depends on the expertise of the physical therapist (PT) and might be inaccurate [11]. A real-time, objective characterization of gait impairments would allow the PTs to provide patient-specific feedback on gait performance to be used during rehabilitation interventions.

Wearable sensor-based systems can be used to help PTs guide feedback in real-time based on objective measures of gait [12]. Feedback-based interventions, using wearable sensors have shown promising results for gait rehabilitation [12] but studies have been limited to treadmill-based systems and lower body-related metrics [12, 13]. We developed *Mobility Rehab* a novel, PT-assisted visual feedback system, for providing real-time measures of upper and lower body gait metrics [14].

The *Mobility Rehab* system uses wireless, inertial sensors (Opals, APDM Wearable Technologies, a Clario company) worn on the wrists, feet, and sternum area to improve the accuracy and effectiveness of PT’s feedback to their patients [14]. The *Mobility Rehab* system provides feedback on 5 gait metrics: step duration, stride length, elevation of feet at mid-swing, arm swing range-of-motion (ROM), and trunk coronal (mediolateral) ROM.

We previously demonstrated that one session of *Mobility Rehab* during treadmill gait training showed significant and moderate − to − large effect sizes (ES) in upper and lower body gait metrics (e.g.; arm swing ROM and foot-strike angle) during overground walking in people with Parkinson’s disease [14]. In addition, participants perceived moderate-to-excellent effects on their gait after using the system and no adverse events were reported [14]. These previous results are promising since a meta-analysis showed only small ES of standard PT training on lower body gait metrics, such as gait speed and stride length [15], and no effect on double-support time [15]. In addition, another meta-analysis revealed that patient-perceived mobility, assessed with the Activities − specific Balance Confidence Scale (ABC) scale, did not show significant effects after PT training alone [16]. Thus, we hypothesized that standard PT combined with *Mobility Rehab* (PT + MR) would be more effective than standard PT gait training alone to improve patient-perceived mobility, gait speed as well as upper and lower body gait metrics. This is a pragmatic clinical trial in an independent outpatient PT clinic that provides standard gait training to older adults and people with neurologic diseases and mobility disturbances.

The objective of this study was to compare the effects of PT + MR versus PT gait training alone, on ABC scores (primary outcome), gait speed (secondary outcome), and exploratory upper and lower body gait metrics (step time asymmetry, foot clearance, arm swing, trunk coronal ROM, and foot strike angle) during overground walking in older adults and people with neurologic diseases in an outpatient PT clinic. We also report the feasibility of using *Mobility Rehab* in an outpatient physical therapy clinic, where time with each patient is limited.

## Methods

### Design

This study is a single-site, pragmatic clinical trial in older people referred to physical therapy for gait impairments. This trial compared the effects of PT + MR versus PT alone on ABC scores and objective measures of overground walking. Intervention sessions and blinded assessments were performed at Northwest Rehabilitation Associates (NWRA), an outpatient rehabilitation center in Salem (Oregon, USA). The study was approved by the Institutional Review Board at Oregon Health & Science University (eIRB # 16,282), registered on ClinicalTrials.gov (NCT03869879) and the protocol was previously published [17].

### Participants

Individuals were recruited from the NWRA. There was no gender, ethnic, or racial minority exclusions for this study. Participants were included in the study if they were able to follow instructions (PT’s judgement), were 60–89 years old, and had gait disturbances with referral to PT. Gait disturbances, for example, slow gait, reduced stride length, shuffling, and decreased arm swing were identified visually by the PT with expertise in gait disturbances. Outpatients with these gait disturbances identified by the PT were referred for gait training at the NWRA clinic. Individuals gave their written informed consent to participate and were instructed on the study’s procedures.

### Procedures and intervention

Participants scheduled at the physical therapy clinic for gait training were assigned to one of the 4 PTs, and if eligible for this study, which determined their group assignment for the duration of their outpatient therapy. Two therapists (one more experienced, > 10 years of practice and one with < 5 years of practice) were trained to use the *Mobility Rehab* system and two therapists (one more experienced, > 10 years of practice and one with < 5 years of practice) carried out standard PT. The participants were assigned to groups depending on PT’s open schedule. Although the patients were not randomly assigned to each group, this approach was practical for the clinic and avoided selective enrollment assignment since the PT aide assigns an upcoming patient to a PT according to schedule availability without regard to diagnosis, severity, age, or sex. Also, unfortunately, the COVID-19 pandemic had a drastic impact on recruitment, which interfered with the assignment and randomization.

The intervention details have been previously published [17]. Briefly, participants in both groups (PT + MR and PT alone) had pre- and -post training assessments using Mobility Lab v2, (upper and lower body assessment with 6 Opal sensors – both wrists and feet, and at the lumbar and sternum level). The *Mobility Rehab* system used during training had five Opal sensors (both wrists and feet, and at the sternum level). Participants trained twice a week for 4 weeks (8 sessions). Sessions were 45 min long and gait was trained for 30 min in each session. The additional 15 min included exercises for endurance, strength, and static and dynamic balance in functional tasks. The difference between the two groups was the use of the *Mobility Rehab* system in the PT-assisted feedback group. The PTs designed their own treatment plan for each patient and were allowed to select modality, overground walking and/or treadmill, and tasks (dual task, head turns, etc. during gait training) as appropriate for each patient. During a typical session, patients worked on improving quality of gait with the following tasks for 30 min: weights on ankles, dual tasks, upper extremity support, partial body weight support, speed challenges, direction changes, navigating obstacles, and head turning. The PTs using *Mobility Rehab* received specific training on how to effectively use the system (e.g., sensors placement, software navigation, selection and interpretation of metrics, etc.) before the start of the trial.

The *Mobility Rehab* system includes a tablet (to visualize gait measures) and five Opal sensors (APDM Wearable Technologies—Clario company, Portland, Oregon, USA), that are placed on both wrists and feet, and at the sternum level, as we have previously published [14]. The *Mobility Rehab* system provides real-time feedback on five gait metrics: step duration, stride length, elevation at mid-swing, arm swing ROM, and trunk coronal ROM. The metrics were selected based on reviews of the literature [13, 18–20]. Additionally, the algorithms producing these measures have been thoroughly validated with optical motion capture and GAITRite mat in different patient populations including Parkinson’s disease, multiple sclerosis, stroke, healthy young and older controls. Finally, we used different metrics as outcomes to promote benefits that go beyond the specificity effects. The PTs selected any of these gait metrics and focused on one or several gait metrics during each intervention session. Visual feedback from the tablet could be provided directly to patients while walking on a treadmill, and/or to the PTs who gave auditory feedback while the patients walked overground. Therapists also selected a minimum and maximum goal for each metric, based on each patient’s abilities.

### Outcome measures

The primary outcome measure was the Activities-specific Balance Confidence (ABC) scale, a patient-reported-outcome measure of subjects’ perceived mobility [21]. The ABC has been shown to reflect the activities in which people actually participate that involve walking [22]. The secondary outcome measure was gait speed [17]. In addition, the following exploratory variables were considered: step time asymmetry, foot clearance, arm swing ROM, trunk coronal ROM, and foot strike angle. We used different metrics as outcomes to promote benefits that go beyond the specificity effects. All these gait metrics were averaged during a 2-minute-long walk performed in two different conditions: (1) comfortable speed, and (2) as fast as possible. At their first therapy session, a PT-aide placed six Opal sensors on the participants’ feet, wrists, lumbar, and sternum areas and participants were instructed to walk back and forth along a 9-m corridor. An immediate report was generated by Mobility Lab v2 (APDM Wearable Technologies) that characterized the participants’ gait with the objective metrics, as previously published [23]. The treating PT determined, from the report and by their clinical judgment, which variables (step duration, stride length, elevation at mid-swing, arm swing ROM, and trunk coronal ROM) to use for the feedback gait training [14]. The post-testing (walking tests at comfortable and fast pace) assessment was performed after the last training session (on the same day).

### Feasibility of using the Mobility Rehab system

Before deploying the system in the clinic, several meetings took place to ensure the *Mobility Rehab* system was easy to use and matched to physical therapist expectations. After those meetings, 10 PTs were requested to try out the system for a week at NWRA, after which they were interviewed to document their experience with the *Mobility Rehab* system. The PTs answered questions about their experience using the visual feedback (such as ‘Are the displayed graphs easy to understand?’, ‘How do you rate the perceived usefulness of the system?’, ‘Can you quickly identify which metric to train?’, ‘Will your patients benefit from the visual feedback after a walking training session?’). The PT participants then completed a Perceived Usefulness and Ease of Use questionnaire and ratings ranged from useful to extremely useful.

### Statistical analysis

To compare the characteristics between groups at baseline, we used independent t-tests. Normality and the presence of extreme observations were assessed through the Shapiro-Wilk test and box-plots, respectively.

As we were not able to achieve our proposed sample size in the protocol paper due to the COVID-19 pandemic and the fact that the clinic (NWRA) was acquired by a larger corporation, the statistical approach evaluated ES.

To test for the effects of PT + MR and PT alone on outcome measures, ES and confidence interval (CI) were calculated for within-group (before vs. after) comparisons [24]. The estimated mean and standard deviation (SD) delta changes from each group were used to calculate ES and CI. The 95% CI of the ESs were calculated using a non-central t distribution [25–27]. Positive and negative CI [i.e., not crossing zero (0)] were considered as significant. The ES has been suggested for within-group comparisons as it allows the determination of the magnitude of the treatment effects and it is well accepted for studies with a small sample size [26]. ESs were classified as small (ES 0.20–0.49), medium (ES 0.50–0.79), and large (ES ≥ 0.80) [28].

## Results

### Participant’ characteristics

**Fig. 1:**
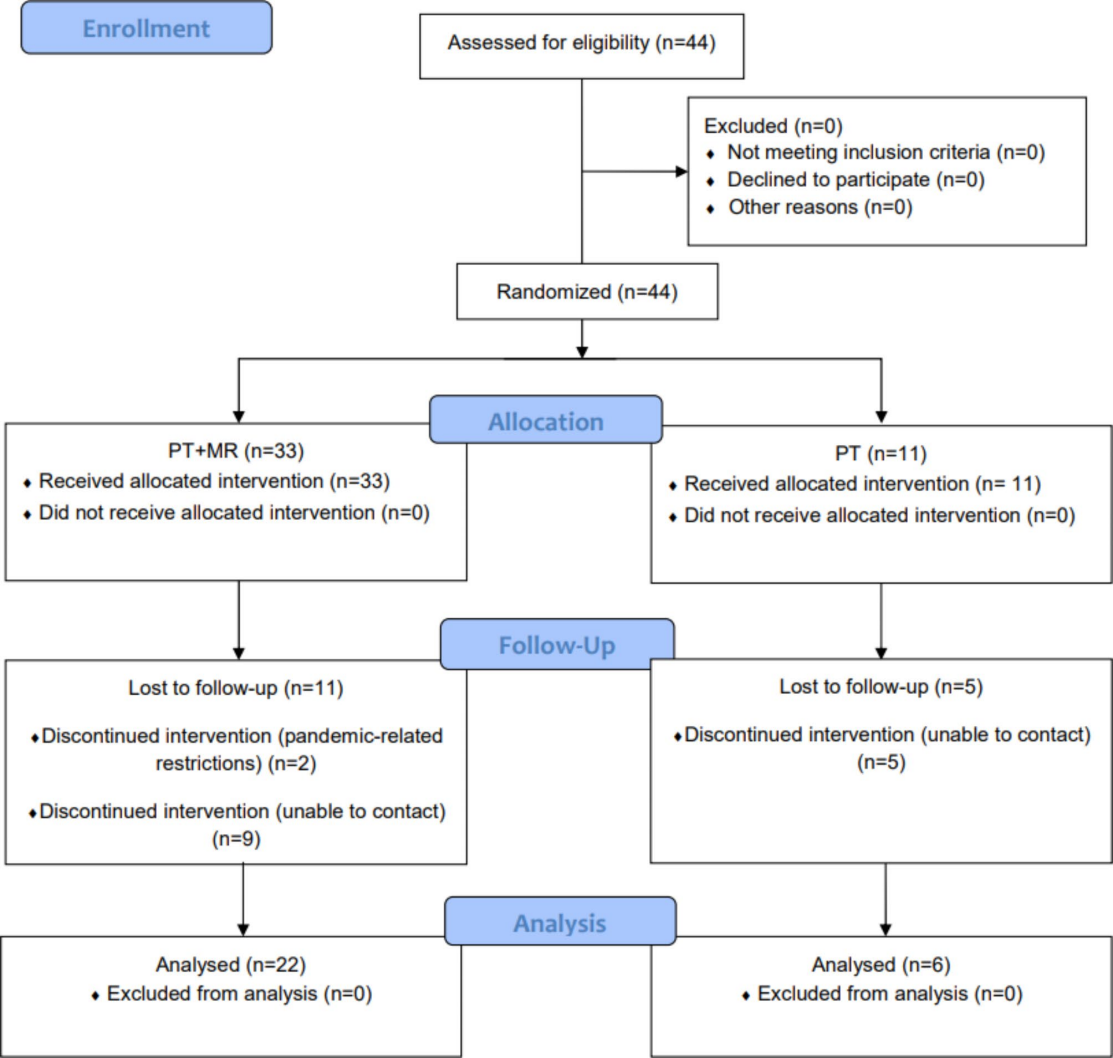
Trial profile with schematic representation of participant recruitment and allocation. PT + MR = Physical therapy with *Mobility Rehab* system; PT = Physical therapy

As previously reported [17], this trial was powered for a total sample size of 200 subjects. However, we had a significant change in number of participants collected for this pragmatic clinical trial. Unfortunately, the COVID-19 pandemic had a drastic impact on both recruitment and follow-up. Patients who were afraid of getting COVID did not continue in the study. Also, as expected, the NWRA closed for some time during the pandemic, and subsequently was bought by a larger PT corporation. This led to a high number of drop-outs, prevented new recruitments, and resulted in a high personnel turnover, who we had to keep trained. At the end, excluding 16 drop-outs, this study evaluated 28 older adults with and without neurological diseases (Fig. 1) who were randomized either to the PT + MR (n = 22) or to the PT alone (n = 6), who completed baseline and follow-up assessments.

### Baseline

There were no between-group differences in any variable (Table 1). The 28 study participants seeking physical therapy for gait abnormalities included 10 older adults (PT + MR = 8; PT = 2), 4 people with Parkinson’s disease (PT + MR = 3; PT = 1), 2 people with multiple sclerosis (PT + MR = 2; PT = 0), 9 post-stroke people (PT + MR = 7; PT = 2), one person with central cord syndrome (PT + MR = 1; PT = 0), one person with spinal cord tumor (PT + MR = 1; PT = 0), and one person with cerebellar ataxia (PT + MR = 0; PT = 1).

**Table 1:** Characteristics of the individuals. Mean(SD) are shown

	PT + MR (n = 22)	PT (n = 6)	*P* value
**Characteristics**			
Men/women (number)	9/13	5/1	
Age (years)	69.6(12.4)	67.7(11.6)	0.592
Exercise per week (hours)	9.2(9.2)	8.7(3.4)	0.501
Disease duration (years)	3.8(2.6)	6.3(8.0)	0.737
Fall in the last 12 months (number)	2.3(1.7)	2.6(1.4)	0.431
Fall in the last 6 months (number)	0.7(1.0)	1.0(1.1)	0.648
**Diagnosis**			
Parkinson’s disease (number)	3	1	
Older adults (number)	8	2	
Multiple Sclerosis (number)	2	0	
Stroke (number)	7	2	
Central Cord Syndrome (number)	1	0	
Spinal Cord Tumor (number)	1	0	
Cerebellar Ataxia (number)	0	1	
**Outcomes**			
ABC (scores)	59.1(23.8)	53.6(21.8)	0.603
*Comfortable pace*			
Gait speed (m/s)	0.7(0.3)	0.8(0.3)	0.407
Step time asymmetry (a.u.)	0.04(0.08)	0.06(0.07)	0.380
Foot clearance (cm)	1.92(0.98)	1.76(0.71)	0.887
Arm swing ROM (degrees)	31.9(16.1)	34.5(10.3)	0.441
Trunk coronal ROM (degrees)	5.6(2.5)	9.7(9.9)	0.744
Foot-strike angle (degrees)	15.6(6.9)	15.2(7.5)	0.798
*Fast pace*			
Gait speed (m/s)	0.8(0.3)	0.9(0.4)	0.628
Step time asymmetry (a.u.)	0.04(0.08)	0.07(0.10)	0.662
Foot clearance (cm)	2.2(1.0)	1.9(0.7)	0.842
Arm swing ROM (degrees)	40.9(21.1)	48.4(28.2)	0.628
Trunk coronal ROM (degrees)	6.1(2.6)	11.3(10.6)	0.457
Foot-strike angle (degrees)	16.8(7.4)	15.9(10.1)	0.762

PT + MR = Physical therapy with *Mobility Rehab* system; PT = Physical therapy; ABC = Activities-specific Balance Confidence Scale scores; ROM = range-of-motion.

### Patient-perceived mobility (primary outcome) did not show significant changes after either intervention

**Fig. 2:**
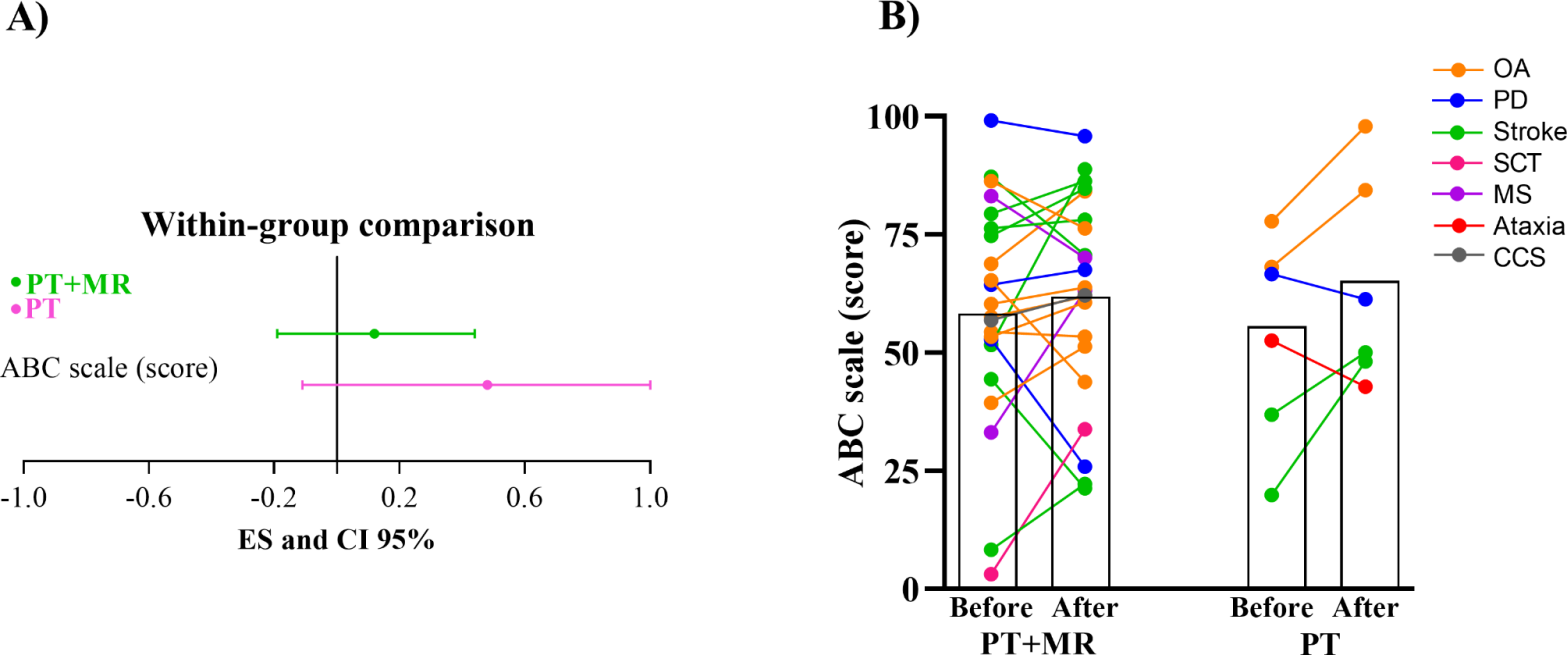
(**A**) Effect size and confidence interval comparisons within group (PT + MR = Physical therapy with *Mobility Rehab* system and PT = Physical therapy) for Activities-specific Balance Confidence Scale (ABC). (**B**) Mean for the ABC scale score before and after 8 sessions of PT + MR and PT in OA: older adults; PD: Parkinson’s disease; SCT: Spinal Cord Tumor; MS: Multiple Sclerosis; CCS: Central Cord Syndrome

PT + MR (ES = 0.12, 95% CI= -0.19 to 0.44) and PT (ES = 0.48, 95% CI= -0.11 to 1.00) showed nonsignificant effects on ABC scores following the 8 sessions of gait training, see Fig. 2 for details.

### PT + MR showed significant effects on gait speed (secondary outcome) during overground walking in comfortable and fast pace

**Fig. 3:**
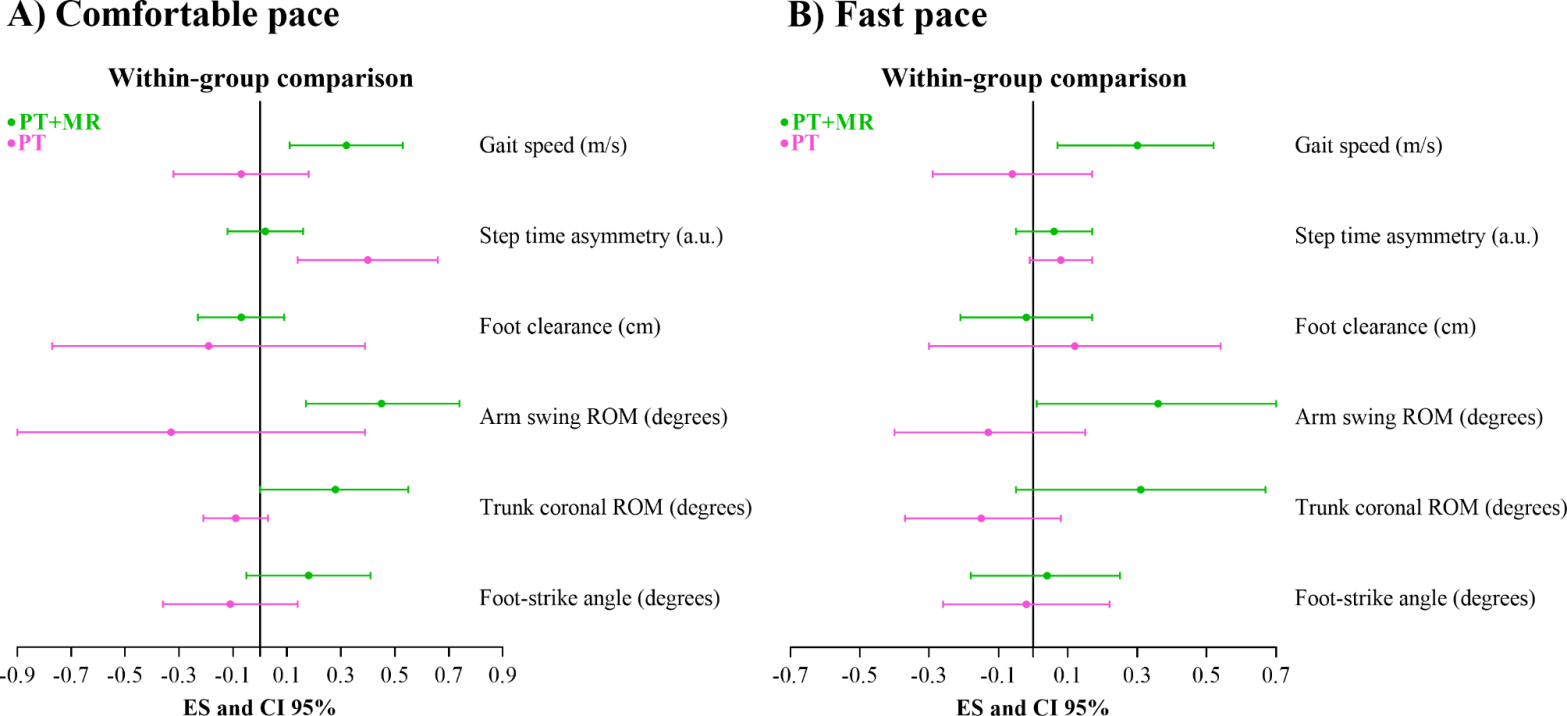
Effect size and confidence interval comparisons within group for gait metrics in comfortable (**A**) and fast pace (**B**). PT + MR = Physical therapy with *Mobility Rehab* system; PT = Physical therapy; ROM = range-of-motion.

**Fig. 4:**
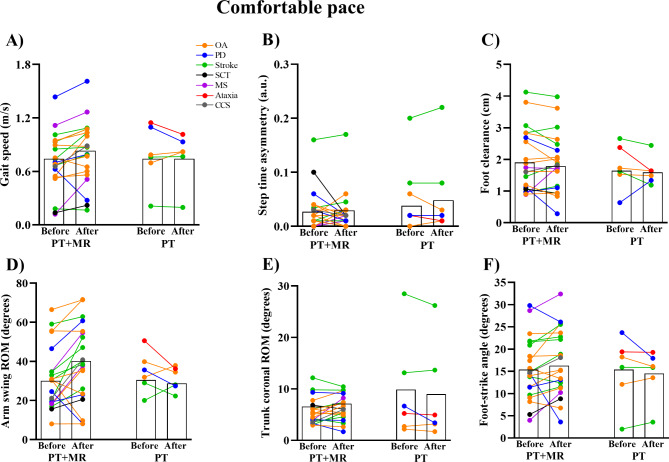
Mean for the gait speed (**A**), step time asymmetry (**B**), foot clearance (**C**), arm swing range-of-motion (ROM) (**D**), trunk coronal ROM (**E**), and foot-strike angle (**F**) in comfortable pace before and after 8 sessions of PT + MR (Physical therapy with *Mobility Rehab* system) and PT (Physical therapy) in OA: older adults; PD: Parkinson’s disease; SCT: Spinal Cord Tumor; MS: Multiple Sclerosis; CCS: Central Cord Syndrome

**Fig. 5:**
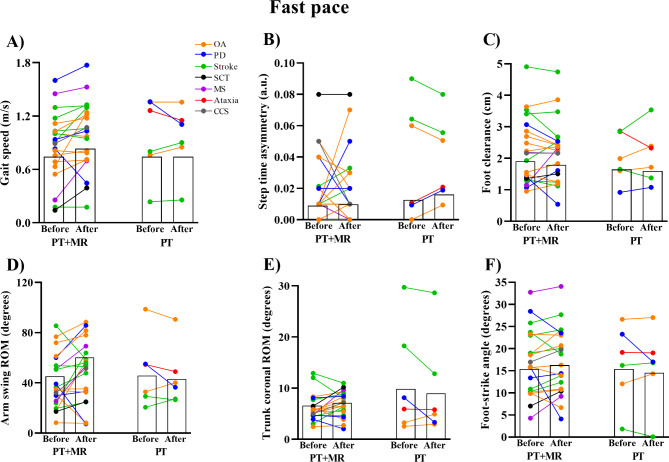
Mean for the gait speed (**A**), step time asymmetry (**B**), foot clearance (**C**), arm swing range-of-motion (ROM) (**D**), trunk coronal ROM (**E**), and foot-strike angle (**F**) in fast pace before and after 8 sessions of PT + MR (Physical therapy with *Mobility Rehab* system) and PT (Physical therapy) in OA: older adults; PD: Parkinson’s disease; SCT: Spinal Cord Tumor; MS: Multiple Sclerosis; CCS: Central Cord Syndrome

PT with *Mobility Rehab* gait training showed small, significant improvements on gait speed during comfortable (ES = 0.32, 95% CI = 0.11 to 0.53) and fast pace (ES = 0.30, 95% CI = 0.07 to 0.52). In contrast, PT gait training, alone, did not result in a significant effect on gait speed during comfortable (ES= -0.07, 95% CI= -0.32 to 0.18) or fast paced gait (ES= -0.06, 95% CI= -0.29 to 0.17), see Figs. 3, 4 and 5 for details.

### PT + MR showed significant effects on specific upper and lower body gait metrics during overground walking

Walking at a comfortable speed. Only PT + MR showed small and significant effects on arm swing ROM (ES = 0.45, 95% CI = 0.17 to 0.74) and trunk coronal ROM (ES = 0.28, 95% CI = 0.00 to 0.55). PT with *Mobility Rehab* showed no effect on step time asymmetry (ES = 0.02, 95% CI= -0.12 to 0.16), foot clearance (ES= -0.07, 95% CI= -0.23 to 0.09), or foot strike angle (ES = 0.18, 95% CI= -0.05 to 0.41). PT alone showed no significant effect on any upper or lower body gait metric (step time asymmetry: ES = 0.40, 95% CI = 0.14 to 0.66; foot clearance: ES= -0.19, 95% CI= -0.77 to 0.39; arm swing ROM: ES= -0.33, 95% CI= -1.11 to 0.39; trunk coronal ROM: ES= -0.09, 95% CI= -0.21 to 0.03; foot strike angle: ES= -0.11, 95% CI= -0.36 to 0.14), see Figs. 3 and 4 for details.

Walking at fast speed. Only PT with *Mobility Rehab* showed small and significant effects on arm swing ROM (ES = 0.36, 95% CI = 0.01 to 0.70), but no effect on foot clearance (ES= -0.02, 95% CI= -0.21 to 0.17), trunk coronal ROM (ES = 0.31, 95% CI= -0.05 to 0.67), step time asymmetry (ES = 0.06, 95% CI= -0.05 to 0.17), and foot strike angle (ES = 0.04, 95% CI= -0.18 to 0.25). PT alone showed no significant effect on upper and lower body gait metrics (step time asymmetry: ES = 0.08, 95% CI= -0.01 to 0.17; foot clearance: ES = 0.12, 95% CI= -0.30 to 0.54; arm swing ROM: ES= -0.13, 95% CI= -0.40 to 0.15; trunk coronal ROM: ES= -0.15, 95% CI= -0.37 to 0.08; foot strike angle: ES= -0.02, 95% CI= -0.26 to 0.22), see Figs. 3 and 5 for details.

### Adherence and adverse events

Adherence to both training protocols was high. Participants who performed PT + MR completed 7.8 ± 0.4 sessions (98%) and participants who performed PT completed 8.0 ± 0 sessions (100%). No adverse event was reported during the trial.

### PT feedback on Mobility Rehab system

All the PT participants answered that they “would like to use the system in their practice’”, “the visualization is useful”, and “patient could benefit from using the system’”. In addition, at the end of the pragmatic trial, the 2 PTs administering the PT + MR intervention were interviewed again. They reported that having the option to provide real time feedback to individuals with gait abnormalities was very beneficial.

In addition, the PTs administering PT + MR reported that individuals using the system enjoyed having quantitative feedback to get a better picture of the asymmetries that were present or other gait abnormalities. Targeted cueing and attention to certain aspects of their gait with ability to see the respective changes on the real time feedback was very motivational for these individuals. As far as system set-up, the PTs reported that the setup of the device was intuitive, didn’t take too much time (less than 5 min), and added to the overall patient experience in improving their mobility and confidence.

## Discussion

To the best of our knowledge, this is the first study to explore the feasibility and effectiveness of a wearable PT-assisted feedback system in an outpatient clinic to older adults with mobility disturbances. This trial was severely impacted by the COVID-19 pandemic that dramatically reduced the enrollment. However, our main findings showed that although patient-reported outcome of mobility did not change after PT nor PT + MR, gait speed, our secondary outcome, and exploratory outcome measures of upper body gait metrics (arm swing and trunk coronal ROM) improved with *Mobility Rehab* but not traditional PT alone.

### Neither intervention improved the patient-reported outcome of mobility

The ABC was used as the primary outcome for this clinical trial because it is reflective of the subjects’ perception of mobility [22]. The ABC scores did not improve with either intervention. Consistent with our study, a meta-analysis showed that the ABC scores did not show significant effects after 7 or 12 weeks of PT training with and without virtual reality [16]. On the other hand, our previous clinical trial showed that a group exercise of 6 weeks of Agility Boot Camp with cognitive challenge showed a small improvement (small ES) on the ABC scores in people with Parkinson’s disease [29]. These results suggest, that to improve perception of mobility, studies should investigate either the effects of longer duration of PT (> 12 weeks) or a more cognitively challenging intervention of shorter duration (~ 6 weeks). Thus, future studies should investigate if longer-duration PT using cognitively-challenging mobility exercises combined with the *Mobility Rehab* system improve subjects’ perception of mobility with gait disturbance in an outpatient clinic that provides standard PT training to older neurological patients with mobility disturbances.

It is important to highlight that the PT group nearly reached a medium effect size and showed a minimal clinically important difference for the ABC scores (10.5%) for patient perceptions about their functional balance ability. A minimal clinically important difference of 10% for the ABC scores has been suggested for outpatients with neurological conditions (e.g., Parkinson’s disease, multiple sclerosis, and stroke) [30]. Our results suggest that a larger sample size would be needed to reach statistical significance despite the minimal clinical improvement of 10.5% on the ABC scores after the PT program. There is limited evidence so far on whether the successful application of feedback-based interventions could be effective in improving patients’ perception of mobility [12], since most of the studies have only focused on objective measures of balance [12]. Thus, future studies with a large sample size are needed to determine whether the effects of the *Mobility Rehab* system during PT training may transfer from trained objective gait metrics to patients’ perception of mobility.

### Mobility Rehab system had positive effect on gait speed

The secondary outcome measure, gait speed, has been reported as the ‘6th vital sign’ and predictive of mortality [31]. Gait speed is frequently used in geriatric settings as a quick, simple, and reliable way of estimating older patients’ functional capacity [32, 33]. Reduced gait speed is a sign of advancing age, and it is associated with poorer response to rehabilitation, age-related diseases, including cardiovascular disease and dementia, and early mortality [34–37]. Gait speed was not directly trained but real-time feedback was provided about quality of upper and/or lower body characteristics of gait, while patients walked overground or on a treadmill [14]. This pilot study showed that PT with *Mobility Rehab*, but not PT alone, had significant but small effect on gait speed in older adults with gait disturbances, with and without neurological diseases. Thus, our results were promising for an outpatient rehabilitation center, such as NWRA, that typically provides services to people with gait disturbances with and without neurological diagnoses (mobility impairments – nonspecific geriatric, Parkinson’s disease, stroke, spinal cord tumor, multiple sclerosis, ataxia, and central cord syndrome, and neuropathy). This pilot study indicates practical effects of PT with *Mobility Rehab* in an outpatient clinic, outside the laboratory conditions.

Our previous study, conducted in laboratory, showed that one session of *Mobility Rehab* during treadmill gait training had significant and moderate − to − large immediate effects on upper and lower body gait metrics during overground walking in people with Parkinson’s disease [14]. In the current pragmatic clinical trial, the participants used the *Mobility Rehab* system imbedded within their regular PT training sessions and included overground walking as well as treadmill gait training, as appropriate. The training was designed by the PT specifically for each patient. Our results are clinically relevant for people with gait disturbances at an outpatient rehabilitation center because faster gait at a comfortable pace (change score of 0.11 m/s) after PT with *Mobility Rehab* system was better than the minimal clinically important difference of 0.10 m/s suggested for patients with neurological conditions (e.g., multiple sclerosis and stroke) [38]. Such changes have not been observed after other types of physical training with wearable sensors as demonstrated in a previous meta-analysis that included 8 randomized controlled trials [12]. These results suggest that the Mobility Rehab system during PT training may have the potential to improve objective gait metrics. Future studies are needed to explore this system with a larger sample size.

### Only the PT with Mobility Rehab system had positive effects on upper and lower body gait metrics

Although gait has stereotypic characteristics with individual differences and significant variability of gait pattern occurs, even among individuals who share the same neurologic diagnosis, general patterns of gait disturbance are associated with common neurological disturbance [3, 5–9]. In diverse populations, gait impairments often manifest as slow gait with decreased arm swing and abnormal (too big or too little) trunk coronal ROM [3, 5–9].

Our pilot results showed possible improvement in lower and upper body gait measures after PT with *Mobility Rehab* but not after PT alone with improvement in gait speed, arm swing ROM and trunk coronal ROM across different conditions. These results are potentially important for people with neurologic disorders in an outpatient clinic, as gait disturbance is a manifestation of a primary problem that alters neural control of ambulation [3, 5–9]. Thus, the *Mobility Rehab* system may be an efficient method to correct this primary problem toward the goal of improving mobility in an outpatient clinic. We did not find any association between gait speed changes and arm swing and trunk ROM changes so the improvement in arm swing and trunk ROM are likely due to the biofeedback training (*Mobility Rehab* system), which would suggest that PT training with the *Mobility Rehab* system may cause improvement in specific mechanisms of motor control.

There is a need to establish the effectiveness of PT-assisted feedback in an actual outpatient therapy setting. Depending on the clinical setting, 40% of patients have gait abnormalities [39]. *Mobility Rehab* provides therapists stride-by-stride, relative measures of gait quality, as well as summary statistics, on a tablet as their patient walks overground in natural conditions so they can provide quick, accurate verbal instructions to their patients in an outpatient clinic. *Mobility Rehab* system has the potential to be used in an outpatient clinic to improve the accuracy and effectiveness of therapists’ feedback to their patients by providing objective measures of gait in real-time.

### Study limitations

This study has several limitations. Although we were able to conduct a pragmatic clinical trial that is more difficult and more relevant than a laboratory study in a controlled environment, the sample size was dramatically lower than anticipated due to the COVID-19 pandemic. Hence, larger and better powered studies should be carried out to confirm these promising and preliminary results. Second, we did not assess subjects in the longer-term, thus, we do not know if the improvements in gait metrics after the 8 sessions were retained. Third, even though our study showed significant changes in gait speed, arm swing ROM and trunk coronal ROM after PT + MR, but not after PT alone, a larger clinical trial is needed to validate the reported benefits of the PT + MR on those exploratory gait metrics in older adults and people with neurological diseases. Fourth, although a PT aide is not needed for the *Mobility Rehab* system to be used, that role was fundamental in this study. In fact, the help of an assistant in setting up the system and placing sensors on the patient was useful to save therapists’ time.

## Conclusions

Physical therapy alone or combined with *Mobility Rehab* did not improve the patient-reported outcome of mobility (ABC score). Only physical therapy combined with *Mobility Rehab* improved gait speed and upper body gait metrics in older adults with and without neurological diseases in an outpatient clinic that provides standard physical therapy training to patients with mobility disturbances.

## Data Availability

The data are available from the corresponding author on reasonable request.
